# Expression Analysis, Diagnostic Significance and Biological Functions of BAG4 in Acute Myeloid Leukemia

**DOI:** 10.3390/medicina61081333

**Published:** 2025-07-24

**Authors:** Osman Akidan, Selçuk Yaman, Serap Ozer Yaman, Sema Misir

**Affiliations:** 1 Department of Hematology, Mengucek Gazi Education and Research Hospital, 24180 Erzincan, Turkey; osmanakidandr@hotmail.com; 2 Department of Medical Biochemistry, Trabzon Kanuni Training and Research Hospital, 61290 Trabzon, Turkey; 3 Department of Medical Biochemistry, Trabzon Kanuni Health Practice and Research Hospital, Trabzon University of Health Sciences, 61290 Trabzon, Turkey; 4 Department of Biochemistry, Faculty of Pharmacy, Sivas Cumhuriyet University, 58140 Siva, Turkey

**Keywords:** acute myeloid leukemia, BAG4, cell cycle

## Abstract

*Background and Objectives*: A thorough comprehension of the essential molecules and related processes underlying the carcinogenesis, proliferation, and recurrence of acute myeloid leukemia (AML) is crucial. This study aimed to investigate the expression levels, diagnostic and prognostic significance and biological roles of Bcl-2-associated athanogene 4 (BAG4) in AML carcinogenesis. *Materials and Methods*: Gene expression profiles were analyzed using publicly available datasets, particularly GSE9476 and TCGA, using tools such as GEO2R, GEPIA2, UALCAN and TIMER2.0. The immune infiltration correlation was examined using the GSCA platform, while the function of BAG4 at the single-cell level was analyzed via CancerSEA. Protein–protein and gene–gene interaction networks were constructed using STRING and GeneMANIA, and enrichment analyses were performed using GO, KEGG and DAVID. Expression validation was performed using RT-qPCR in HL-60 (AML) and HaCaT (normal) cells, and ROC curve analysis evaluated the diagnostic accuracy. *Results*: BAG4 was significantly overexpressed in AML tissues and cell lines compared with healthy controls. High BAG4 expression was associated with poor overall survival and strong diagnostic power (AUC = 0.944). BAG4 was positively associated with immune cell infiltration and negatively associated with CD4+/CD8+ T and NK cells. At the single-cell level, BAG4 was associated with proliferation, invasion, and DNA repair functions. Functional network analysis showed that BAG4 interacted with apoptosis and necroptosis-related genes such as BCL2, BAG3 and TNFRSF1A and was enriched in pathways such as NF-κB, TNF signaling and apoptosis. *Conclusions*: BAG4 is overexpressed in AML and is associated with adverse clinical outcomes and immune modulation. It may play an important role in leukemogenesis by affecting apoptotic resistance and immune evasion. BAG4 has potential as a diagnostic biomarker and treatment target in AML, but further in vivo and clinical validation is needed.

## 1. Introduction

Acute myeloid leukemia (AML) is a type of hematologic cancer characterized by the abnormal expansion and accumulation of immature myeloid precursor cells within the bone marrow [1]. AML is the most common form of acute leukemia in adults [2]. The five-year survival rate for patients under 60 is approximately 40%, whereas for those over 60, it drops to below 10–20% [3]. AML is a heterogeneous disease with distinct molecular and clinical subtypes [4], and its prognosis and survival outcomes vary depending on factors such as age, overall health status and genetic and epigenetic variations [5,6]. The primary treatment approaches for AML include chemotherapy and hematopoietic stem cell transplantation; however, high toxicity levels and the risk of relapse significantly limit treatment success [6,7]. Only 40–50% of younger adults and 10–20% of elderly patients achieve long-term survival following treatment [8]. Due to the clinical, morphological, cytogenetic and molecular heterogeneity of AML, these factors play a crucial role in prognosis, enable the incorporation of targeted therapies and guide optimal treatment strategies across different AML subtypes [9]. Consequently, identifying novel biomarkers and potential therapeutic targets is essential for improving the diagnosis and treatment of AML.

BCL-2-associated athanogene 4 (BAG4), also known as the silencer of death domains (SODD), is a member of the BCL-2-associated athanogene (BAG) family [10]. Members of the BAG family (BAG1-6) primarily interact with heat shock protein 70 (HSP70) to regulate its activity; however, their oncogenic properties are becoming increasingly evident. BAG family proteins regulate various interconnected physiological processes, including autophagy, apoptosis and protein homeostasis [11]. BAG4 has been identified as a tumor marker for multiple malignancies and is implicated in tumor progression and drug resistance [12]. Overexpression of BAG4 has been reported in pancreatic [13], ovarian [14] and breast cancers [15], as well as in acute lymphoblastic leukemia [16] and colon cancer [17]. Similar to other oncogenes, BAG4 upregulation typically inhibits tumor cell apoptosis, whereas its downregulation is associated with reduced tumor growth. Consequently, targeting BAG overexpression proteins or inhibiting specific BAG–protein interactions may hold significant therapeutic potential [11]. Anti-apoptotic BCL-2 proteins play a critical role in the survival and drug resistance of AML cells, including AML stem/progenitor cells, suggesting that they may serve as relevant therapeutic targets [18]. Although the existing literature suggests that BAG4 promotes cell proliferation, migration and invasion in various cancers [4,12], its clinical significance and underlying molecular mechanisms in AML remain unclear.

In recent years, tumor bioinformatics has been crucial in cancer research [19]. Bioinformatics analyses provide valuable insights into the biological processes underlying diseases, facilitate the exploration of personalized treatment strategies and prognosis and aid in identifying molecular targets for novel therapies [19,20]. Advanced algorithms enable the analysis of complex datasets, allowing for identifying key genetic markers with significant implications for the diagnosis and treatment of various diseases [20].

This study aims to investigate the expression profile, prognostic significance and functional roles of BAG4 in AML through various bioinformatics and in vitro analyses. By elucidating these aspects, BAG4 may be considered a potential therapeutic target in AML, contributing to development of novel treatment strategies.

## 2. Materials and Methods

### 2.1. Data Collection and Identification of Differentially Expressed Genes (DEGs)

Transcriptomic data corresponding to the GSE9476 dataset, generated using the GPL96 platform (Affymetrix Human Genome U133A Array; Affymetrix Inc., Santa Clara, CA, USA), were retrieved from the Gene Expression Omnibus (GEO) database. The GSE9476 dataset was preferred for this study because it contains aberrant expression changes in AML. This dataset comprises a total of 64 biological samples, consisting of leukemic blast cells from 26 individuals diagnosed with AML and hematopoietic cells from 38 healthy donors serving as controls [21]. DEGs between AML samples and healthy normal hematopoietic cells were identified using GEO2R (http://www.ncbi.nlm.nih.gov/geo/geo2r, accessed on 30 January 2025) [22]. |log2FC|> 1 and *p* < 0.01 were considered statistically significant.

### 2.2. Analysis of BAG4 Expression in Tumor and Normal Tissues

The TIMER2 (Tumor Immunity Prediction Resource, version 2) website (http://timer.cistrome.org/) (accessed on 30 January 2025) was used to investigate the BAG4 expression differences between tumor and normal tissues using data from TCGA [23]. Gene Expression Profiling Interactive Analysis, version 2 (GEPIA2) (http://gepia.cancer-pku.cn/) (accessed on 30 January 2025), was used to obtain BAG4 expression and clinical data from TCGA databases [24]. We used GEPIA2 to examine BAG4 gene expression in AML. UALCAN was used to evaluate BAG4 protein expression in AML [25]. *p* value < 0.05 was recognized.

### 2.3. Diagnostic and Prognostic Value of BAG4

To assess the prognostic value of BAG4 in AML, overall survival (OS) data were obtained from the GEPIA2 and UALCAN platform. Receiver operating characteristic (ROC) curve analysis was performed to evaluate the diagnostic performance of BAG4 in distinguishing AML from healthy cases (GSE9476). The diagnostic efficiency can be summarized using the area under the curve (AUC) of the receiver operating characteristic (ROC) curve.

### 2.4. Correlation Between BAG4 Expression and Immune Infiltration

GSCA (http://bioinfo.life.hust.edu.cn/GSCA) (accessed on 10 February 2025) analyzes cancer gene sets at genomic, pharmacological and immune genomic levels and investigates immune infiltration, gene expression, genomic variation, and gene set expression/mutation [26,27]. The GSCA was utilized to investigate the correlation between BAG4 expression and infiltration of immune cells infiltration in AML. The analysis includes B cells, CD4+ T cells, CD8+ T cells, neutrophils, macrophages, cytotoxic T cells, natural killer (NK) cells, monocytes and dendritic cells (DCs). Spearman’s correlation analysis investigated the correlation between BAG4 expression and immune cell infiltration.

### 2.5. Single-Cell Function in Cancer

The Cancer Single-cell Atlas (CancerSEA) database (http://biocc.hrbmu.edu.cn/CancerSEA/) (accessed on 6 March 2025) enables the analysis of gene functionality at the single-cell level, eliminating the limitations associated with conventional gene expression analysis methods [28].

### 2.6. Interaction Network Analysis of BAG4 and Functional Enrichment Analysis

To investigate the protein–protein interactions (PPIs) involving BAG4, the STRING database (https://cn.string-db.org/) (accessed on 10 February 2025) was utilized to construct an interaction network [29]. Additionally, GeneMANIA (http://genemania.org/) (accessed on 10 February 2025), a comprehensive web-based tool for functional analysis of gene sets, was employed to generate a gene–gene interaction network [30]. Functional annotation of BAG4-related genes was performed using resources from the Gene Ontology (GO) database (http://geneontology.org/) (accessed on 10 February 2025) [31], which classified genes into three principal categories: biological process (BP), cellular component (CC), and molecular function (MF). Moreover, the Kyoto Encyclopedia of Genes and Genomes (KEGG) was consulted for pathway enrichment analysis [32]. To further explore the biological relevance of selected genes—including BAG4, BAG5, BAG3, TNFRSF1A, HSF1 and BCL2—the Database for Annotation, Visualization and Integrated Discovery (DAVID, version 6.8; https://david.ncifcrf.gov/) (accessed on 10 February 2025) was employed to conduct both GO annotation and KEGG pathway enrichment analyses [33]. The cut-off value was set as *p* < 0.05.

### 2.7. Cell Culture and RT–qPCR

HL-60 (CCL-240) and human keratinocyte cell (HaCaT) cells from American Type Culture Collection (Rockville, MD, USA) were seeded in 25 cm^2^ flasks and maintained in a suspension with RPMI-1640 medium enriched with 10% fetal calf serum (FCS) inactivated by heat, penicillin of 192 U/mL and streptomycin of 200 µg/mL concentrations. The cells were incubated in a humidified air atmosphere with 5% CO_2_ at 37 °C. According to the manufacturer’s recommendations, ribonucleic acid was isolated from HL-60 and HaCaT cells using TriReagent (Sigma Aldrich, St. Louis, MI, USA). For the reverse transcription process, complementary DNA (cDNA) synthesis was carried out using the High-Capacity cDNA Reverse Transcription Kit (Applied Biosystems, Thermo Fisher Scientific, Waltham, MA, USA). Quantitative real-time PCR (qRT–PCR) was subsequently conducted employing the PowerUp SYBR Green Master Mix (Applied Biosystems, Thermo Fisher Scientific, Waltham, MA, USA). The primer sequences utilized for amplification of BAG4 and the housekeeping gene GAPDH were as follows: BAG4 forward: 5′-AATGGAGCGTATGGTCCAACA-3′; reverse: 5′-GGCCAAGAGTGTGCTAAAGAA-3′ GAPDH forward: 5′-CAAGGCCAACCGCGAGAA-3′; reverse: 5′-CCCTCGTAGATGGGCACAGT-3′. Quantification of gene expression levels was determined using the comparative threshold cycle (Ct) method, specifically the 2^–ΔΔCt^ approach [34].

### 2.8. Statistical Analysis

The differential expression of the BAG4 gene between HL-60 and HaCaT cell lines was evaluated using an independent samples *t*-test, conducted with IBM SPSS Statistics for Windows (version 23.0; IBM Corp., Armonk, NY, USA). To assess the diagnostic potential of BAG4, a receiver operating characteristic (ROC) curve was generated. ROC curve analysis was performed using MedCalc Statistical Software (version 19.1; MedCalc Software BVBA, Belgium). Statistical significance was set at *p* < 0.05.

## 3. Results

**Figure 1:**
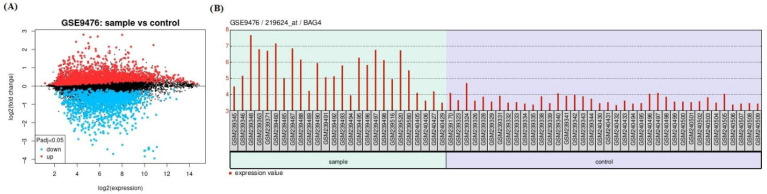
(**A**) Volcano plots show several DEGs differentially expressed in AML compared to normal controls. (**B**) Box plots illustrating the normalized expression levels of BAG4 were generated. The *x*-axis denotes the individual samples, while the *y*-axis represents the corresponding gene expression values.

Firstly, a comparison between AML and normal control groups was performed with GEO2R to identify DEGs from GSE9476. DEGs were identified between AML and normal control groups in both datasets, and the volcano plot visually shows DEGs, as shown in Figure 1A. The expression graph of BAG4 in patients and controls is shown in Figure 1.

**Figure 2:**
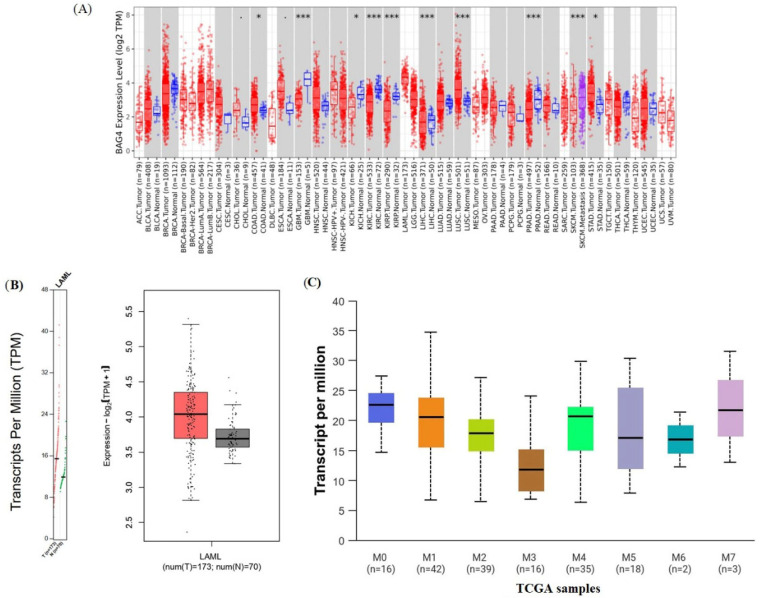
(**A**) Expression distribution of BAG4 gene between tumor (red) and normal samples (blue) * *p* < 0:05, *** *p* < 0:001. (**B**) Box plot of BAG4 gene in AML analyzed using GEPIA2 (red: cancer, gray: normal samples). (**C**) Comparison of BAG4 expression levels among different subtypes of AML based on FAB classifications, analyzed by UALCAN. (LAML = AML).

The same expression trend was consistent with the results obtained from TIMER and GEPIA databases (Figure 2). Also, BAG4 protein levels were determined according to the French American British (FAB) classification in AML (Figure 2). In Figure 2A, the control group represents normal hematopoietic tissues derived from the GTEx dataset, as incorporated into GEPIA2. This analysis unequivocally illustrates increased BAG4 expression in LAML samples compared to normal controls.

Despite the partial overlap of standard deviation bars in Figure 2B, statistical analysis using GEPIA2 verifies a significant difference in BAG4 expression between AML and normal samples (*p* = 0.0001).

**Figure 3:**
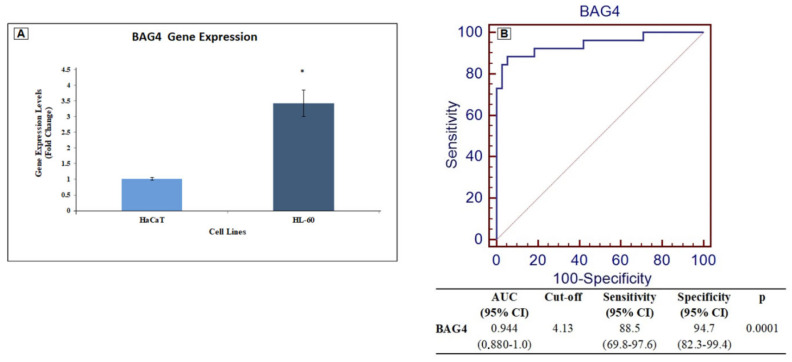
(**A**) The expression level of the BAG4 gene was quantified using RT-qPCR in HL-60 and HaCaT cell lines. All expression data are reported as fold changes relative to HaCaT cells, which served as the control. Gene expression was normalized to GAPDH. A statistically significant difference compared to HaCaT cells is indicated by * *p* < 0.05. (**B**) ROC curve analysis was performed to evaluate the diagnostic performance of BAG4 expression.

According to the RT-qPCR results, BAG4 expression levels were increased in HL-60 compared to HaCaT cells (*p* = 0.0001) (Figure 3A).

ROC analysis was then performed to determine the power of GSE9476 to distinguish AML patients from controls based on the expression levels of this gene. ROC analysis was performed to determine the diagnostic power of BAG4. Accordingly, the ROC analysis results for BAG4 indicated high discrimination power for patients with AML, and the cut-off of value was 4.13 (Figure 3B).

**Figure 4:**
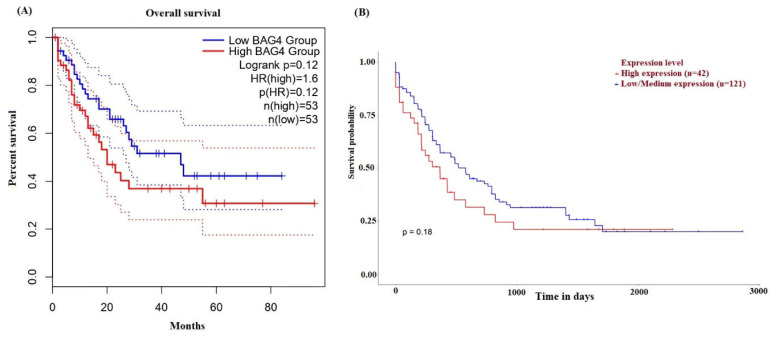
The prognostic significance of BAG4 in AML based on data from the GEPIA2 (**A**) and the UALCAN database (**B**).

To investigate the effect of BAG4 on survival rates AML GEPIA2 and UALCAN databases were used. High expression of BAG4 is an unfavorable prognosis in AML patients. This highlights the significant prognostic value of BAG4 in AML. The OS analysis of BAG4 is shown in Figure 4. For the survival analysis shown in Figure 4A, the statistical comparison between high and low BAG4 expression groups was performed using the log-rank (Mantel–Cox) test, as implemented in the GEPIA2 platform. The findings indicated that elevated BAG4 expression was substantially correlated with reduced overall survival in AML patients (*p* < 0.05). On the other hand, patients with increased BAG4 expression showed a similar tendency of decreased survival in the survival curve derived from the UALCAN database (Figure 4B); however, this difference did not achieve statistical significance (*p* > 0.05).

**Figure 5:**
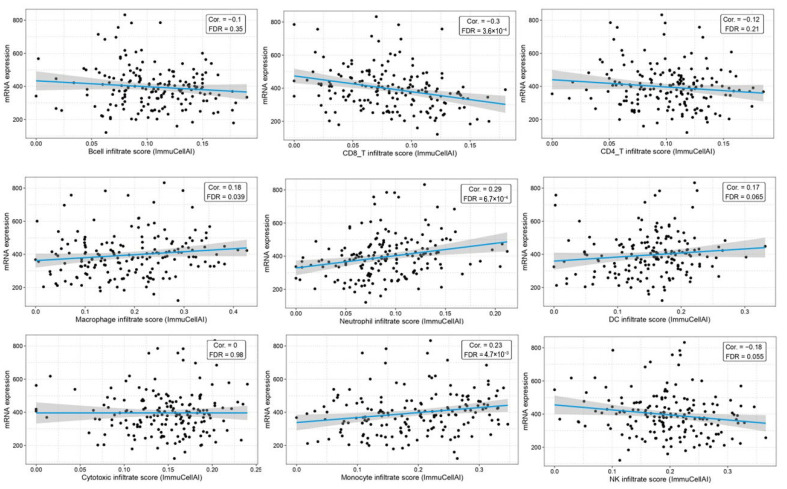
Correlation between BAG4 gene expression and immune infiltration in AML. Scatter plots showing the differences in infiltration levels of B Cells, CD8+ T cells, CD4+ T cells, macrophages, neutrophils and DCs based on BAG4 expression.

With the GSCA tool, we analyzed the correlations between BAG4 expression and immune cell infiltration levels in AML patients. BAG4 expression was negatively correlated with B Cells, CD8+ T cells, CD4+ T cells and NK cells, while it was positively correlated with neutrophils, DCs, macrophages, cytotoxic T cells and monocytes (Figure 5).

**Figure 6:**
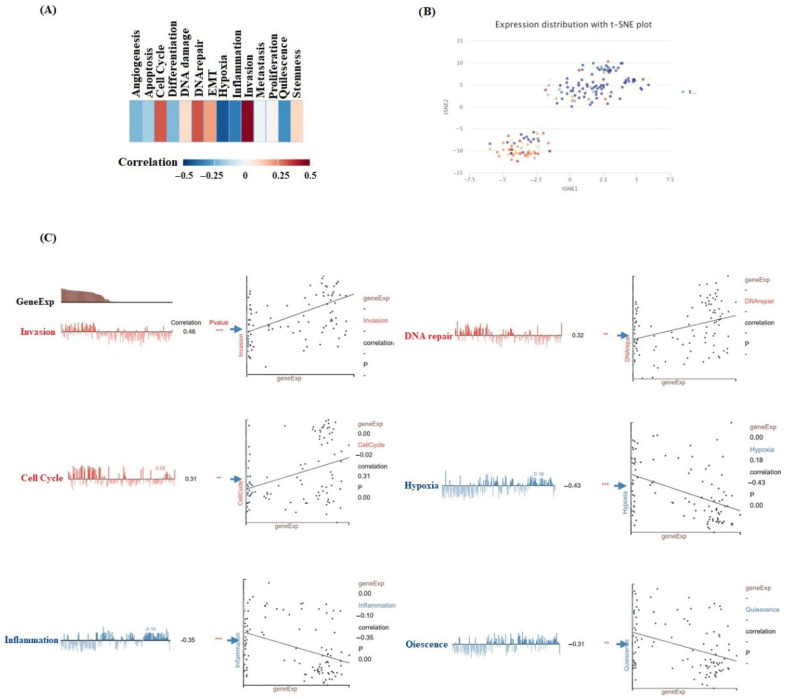
Single-cell expression analysis of BAG4 in AML. (**A**) The association between BAG4 expression and various functional cellular states in AML was evaluated. (**B**) A t-distributed stochastic neighbor embedding (t-SNE) plot was used to visualize the expression patterns of BAG4 at the single-cell level in AML samples. (**C**) Functional correlation analysis further elucidated the detailed relationships between BAG4 expression and specific cellular processes in AML. ** *p* < 0.01; *** *p* < 0.001.

A strong association between BAG4 and AML has been identified. In AML, BAG4 expression positively correlates with four functional states: invasion, cell cycle progression, and DNA repair. Conversely, it negatively correlates with hypoxia, inflammation and quiescence (Figure 6C). Furthermore, analysis using t-SNE has demonstrated that cells with high BAG4 expression tend to form distinct clusters.

**Figure 7:**
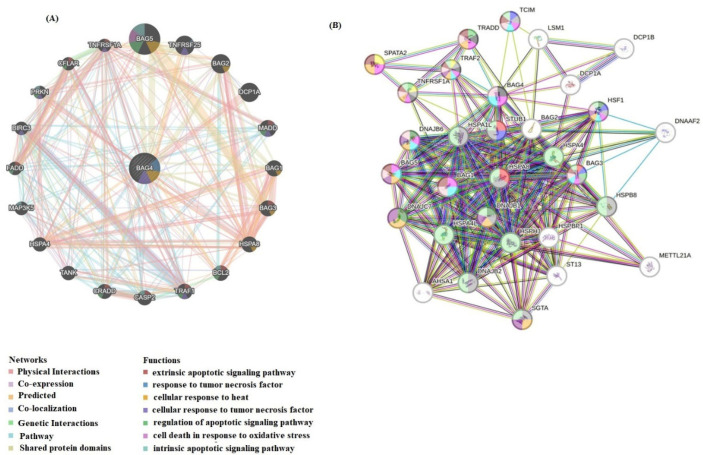
(**A**) The gene–gene functional interaction network of BAG4 generated through GeneMania. (**B**) Protein–protein interaction analysis of BAG4 was demonstrated using STRING. Colors represent different functions. Pink: regulation of programmed cell death, turquoise: negative regulation of apoptotic process, purple: regulation of cellular response to stress, light pink: regulation of apoptotic process, brown: cell death, light green: response to stress, red: chaperone-mediated autophagy.

The PPI network of BAG4-related target genes was obtained from the GeneMANIA and STRING databases (Figure 7A,B). The STRING database demonstrated that the BAG4 gene primarily interacts with BAG1, BAG3, BAG5, DNAJB6, TNFRSF1A, TRADD, TCIM, DNAJB1 and TRAF2. GeneMANIA demonstrated that BAG4 interacts with BAG5, TRAF1, BAG3, BCL2, CFLAR, CASP2, MADD and FADD. Among the genes, especially BAG4, BAG5, BAG3, TNFRSF1A, HSF1 and BCL2 genes, which are related to apoptosis and necroptosis, were selected.

Functional annotation of the co-expressed gene network was conducted using the DAVID bioinformatics tool. The enriched biological processes primarily included the extrinsic apoptotic signaling pathway mediated by death domain receptors, protein folding, suppression of mitochondrial protein targeting, protein stabilization, cellular response to unfolded proteins, inhibition of apoptosis, ligand-independent activation of the extrinsic apoptotic pathway and the intrinsic apoptotic pathway triggered by DNA damage. Additional enriched processes involved cellular responses to heat and mechanical stimuli, regulation of tyrosine phosphorylation of STAT proteins, enhancement of peptidyl-serine phosphorylation and protein localization to the plasma membrane (Table 1). KEGG pathway enrichment analysis indicated that these genes were predominantly associated with key signaling pathways such as apoptosis (across multiple species), NF-kappa B signaling, TNF signaling, sphingolipid signaling, necroptosis and lipid metabolism, including pathways involved in atherosclerosis (Table 2).

## 4. Discussion

Despite significant advancements in understanding the molecular mechanisms underlying AML development [35,36], the disease continues to be associated with a poor prognosis [7]. AML encompasses a complex and heterogeneous disease characterized by various distinct genetic abnormalities. It is frequently associated with an immunologically active (so-called “immune-hot”) tumor microenvironment, which is marked by elevated inflammatory signatures and increased T cell infiltration [3]. Research has demonstrated that numerous genetic abnormalities identified in AML patients are closely linked to disease prognosis [37]. The pathogenesis AML is influenced by multiple factors, including genetic signaling pathways, somatic mutations, and the complex interactions between malignant cells and their microenvironment [38]. To improve patient outcomes, it is essential to identify novel therapeutic targets for AML. Therefore, the characterization of specific oncogenes or signaling molecules associated with AML could facilitate the advancement of molecularly targeted therapies and the discovery of new therapeutic strategies [37].

The BAG (1-6) family of proteins regulates various interconnected physiological processes, including autophagy, apoptosis, and protein homeostasis [11]. Studies have indicated that BAG family proteins exhibit distinct expression variations across different cancer types and tumor cell lines, particularly in leukemias as well as breast, prostate and colon cancers [39]. BAG4 is known to associate with BCL-2 physically, and, through its BAG domain, binds to tumor necrosis factor receptor type 1 (TNF-R1) monomers, thereby inhibiting TNF-alpha-induced apoptosis [4]. Although BAG4 is believed to be involved in the pathology of various cancer types, its specific mechanisms remain inadequately defined [12]. Furthermore, the clinical significance of BAG4 in AML and its potential contributions to molecular pathways have yet to be fully elucidated.

This study aims to investigate the expression dynamics of BAG4, its prognostic relevance, immune infiltration patterns, associated protein and gene interaction networks and its involvement in molecular functions. In our study, publicly available databases such as TIMER2.0, UALCAN and TCGA were utilized to investigate the expression of BAG4 and its prognostic significance in AML. The findings revealed that BAG4 expression varies across different cancer types and is notably elevated in AML (Figure 2A). Additionally, experiments conducted on HL-60 and HaCaT cells demonstrated that BAG4 expression was significantly higher in HL-60 cells than controls, aligning with the database-derived data. A review of the literature on BAG4 highlights several relevant studies. Deng et al. identified BAG4 as a direct target of miR-1-3p, playing a key role in BEZ235-induced apoptosis. The dual PI3K/mTOR inhibitor BEZ235 was found to upregulate miR-1-3p, thereby negatively regulating the expression of BAG4, END1 and ABCB1, ultimately inhibiting cell proliferation and migration while enhancing chemosensitivity [4].

Studies on acute lymphoblastic leukemia (ALL) have also demonstrated that BAG4 is overexpressed in ALL, where it plays a significant role in biological processes such as apoptosis and chemotherapy sensitivity [16,40]. Furthermore, in lung cancer, BAG4 has been implicated in promoting proliferation, invasion, metastasis and drug resistance, functioning within the PI3K/PDK1/AKT and RAF/MEK/ERK signaling pathways [12]. BAG4 has been found to be overexpressed in pancreatic cancer and positively correlated with ANXA7, HSP70 and BCL-2. The BAG4-ANXA7 complex inhibited mitochondrial cytochrome c release and caspase-3 activation, further supporting its role in cancer progression [11].

Protein and mRNA expression profiles can aid in identifying novel biomarkers for cancer diagnosis, facilitating early detection and improving treatment strategies for various human malignancies [41]. To determine whether BAG4 could serve as a biomarker for AML, ROC analysis was conducted. The results demonstrated that BAG4 exhibited a significantly high AUC value (AUC = 0.944, *p* = 0.0001) (Figure 3B). Based on this finding, although validation in a larger cohort is required, BAG4 levels may be a reliable biomarker candidate for AML evaluation. Furthermore, AML patients with high BAG4 expression were observed to have poor OS. Overexpression of BAG4 has been identified as an independent prognostic factor for survival in AML patients. Additionally, previous studies have reported that BAG4 is associated with poor prognosis in various tumor types, including pancreatic, ovarian and breast cancers [4].

Immune cells within the tumor microenvironment (TME) play critical roles in tumor formation. It has been suggested that tumor-associated immune cells may exhibit anti-tumor or pro-tumor functions [42]. The interaction between tumors and the immune system plays a pivotal role in cancer development, progression and treatment. Therefore, clarifying the interactions between tumors and immune cells will aid in predicting responses to immunotherapy and in the development of new immunotherapeutic targets [43]. In recent years, the significance of tumor immunology in AML pathogenesis has increased, with a growing number of studies focusing on immune inhibitory molecules and leukemic antigens [5]. Our study observed a positive correlation between BAG4 expression and neutrophils, DCs, macrophages, cytotoxic T cells and monocytes. In contrast, a negative correlation was found with B cells, CD8+ T cells, CD4+ T cells and NK cells. These results indicate that BAG4 expression is closely associated with immune cell infiltration and suggests that BAG4 may play a significant role in the immune microenvironment of AML.

Protein and gene networks were constructed to elucidate the biological functions of BAG4 and its contribution to molecular regulatory mechanisms. Among these genes, five central genes particularly associated with apoptosis and necrosis were identified: BAG3, BAG5, TNFRSF1A, HSF1 and BCL2. BAG3 is a protein that links apoptosis to autophagy, and it has been noted to be associated with invasion and chemotherapy resistance. The primary function of BAG5 is to act as a nucleotide exchange factor for chaperone proteins. BAG5 is overexpressed in several cancer types and has been linked to poor prognosis in cancer patients [11]. TNFRSF1A, one of the receptors for TNF-alpha, supports tumor development by activating NF-kappaB and regulating inflammation. Additionally, TNFRSF1A plays crucial roles in tumor cell proliferation, invasion and metastasis [44]. Heat Shock Factor 1 (HSF1) is the primary regulator of heat shock response signaling [45]. HSF1 is involved in various processes, such as migration, invasion and proliferation in many cancers [46]. Members of the BCL-2 protein family are critical regulators of apoptotic cell death. Dysregulated expression, characterized either by the overexpression of anti-apoptotic BCL-2 proteins or the downregulation of pro-apoptotic counterparts, leads to the inhibition of apoptosis. Such alterations are commonly observed in various malignancies [47]. Using GO and KEGG pathway enrichment analyses, we found that BAG4 and the genes BAG3, BAG5, TNFRSF1A, HSF1 and BCL2 are concentrated in processes such as regulation of apoptotic signaling pathways, protein folding, negative regulation of mitochondrion-targeted proteins and response to DNA damage. The genes were predominantly enriched in apoptosis-related pathways, NF-kappa B signaling, TNF signaling, necroptosis, lipid metabolism and atherosclerosis. Considering all the results, it appears that BAG4 plays a role in various biological processes, not only through the death receptor-mediated (extrinsic) pathway but also within the mitochondrial (intrinsic) pathway. The upregulation of BAG4 is generally associated with the inhibition of apoptosis in tumor cells, while its downregulation may lead to a reduction in tumor development.

This study has several limitations. The AML patient and control groups were obtained from publicly available databases based on a limited number of individuals. A more extensive and homogeneous cohort study is required. While the potential impact of BAG4 on tumor formation was analyzed through bioinformatics approaches, validation is necessary for the correlations between BAG4 expression, AML prognosis, and immune cell infiltration. Furthermore, although these findings provide new perspectives for future research, further analyses are needed to explore all potential molecular mechanisms of BAG4 in the pathogenesis of AML.

## 5. Conclusions

This study demonstrated that BAG4 is overexpressed in AML samples and cells and is associated with pathological factors that could play a significant role in the development of AML. These findings suggest that BAG4 could serve as an effective biomarker and a potential therapeutic target for AML. BAG4 may contribute to AML progression through multiple mechanisms, including the regulation of apoptotic pathways, drug resistance and immune cell infiltration. Targeting BAG4 is proposed as a novel therapeutic strategy for treating AML and other malignancies in which BAG4 is overexpressed. However, further studies are required to validate these predictive results.

## Figures and Tables

**Table 1:** GO terms enriched by BAG4, BAG5, BAG3, TNFRSF1A, HSF1 and BCL2 genes.

Category	Term	*p*-Value
GOTERM_BP_DIRECT	extrinsic apoptotic signaling pathway via death domain receptors	4.5 × 10^−5^
	protein folding	7.8 × 10^−4^
	negative regulation of protein targeting to mitochondrion	1.5 × 10^−3^
	protein stabilization	1.6 × 10^−3^
	cellular response to unfolded protein	4.6 × 10^−3^
	negative regulation of apoptotic process	6.4 × 10^−3^
	extrinsic apoptotic signaling pathway in absence of ligand	8.7 × 10^−3^
	intrinsic apoptotic signaling pathway in response to DNA damage	1.3 × 10^−2^
	cellular response to heat	1.5 × 10^−2^
	positive regulation of tyrosine phosphorylation of STAT protein	1.6 × 10^−2^
	positive regulation of peptidyl-serine phosphorylation	1.8 × 10^−2^
	cellular response to mechanical stimulus	1.9 × 10^−2^
	protein localization to plasma membrane	4 × 10^−2^
GOTERM_CC_DIRECT	protein folding chaperone complex	7.5 × 10^−3^
	membrane	1.9 × 10^−2^
	cytosol	2.1 × 10^−2^
	cytoplasm	2.5 × 10^−2^
	nucleus	3 × 10^−2^
GOTERM_MF_DIRECT	adenyl-nucleotide exchange factor activity	5.7 × 10^−6^
	protein-folding chaperone binding	3 × 10^−4^
	ubiquitin protein ligase binding	2.5 × 10^−3^
	sequence-specific DNA binding	8.3 × 10^−2^
	protein-containing complex binding	8.7 × 10^−2^
	protein heterodimerization activity	9.7 × 10^−2^

Gene ontology (GO), molecular function (MF), biological process (BP), cellular component (CC).

**Table 2:** KEGG pathway enrichment analysis of BAG4, BAG5, BAG3, TNFRSF1A, HSF1x and BCL2 genes.

Term	Count	*p*-Value
Apoptosis multiple species	2	1.1 × 10^−2^
NF-kappa B signaling pathway	2	3.5 × 10^−2^
Toxoplasmosis	2	3.7 × 10^−2^
TNF signaling pathway	2	4 × 10^−2^
Sphingolipid signaling pathway	2	4.1 × 10^−2^
Apoptosis	2	4.5 × 10^−2^
Fluid shear stress and atherosclerosis	2	4.7 × 10^−2^
Necroptosis	2	5.3 × 10^−2^
Tuberculosis	2	6 × 10^−2^
Human immunodeficiency virus 1 infection	2	7 × 10^−2^
Lipid and atherosclerosis	2	7.1 × 10^−2^
Shigellosis	2	8.2 × 10^−2^
Salmonella infection	2	8.3 × 10^−2^

Count: enriched gene number in the KEGG.

## Data Availability

All bioinformatics data supporting the findings of this study are presented within the article. Data are available from the author upon request.

## Abbreviations

AML	Acute myeloid leukemia
BAG4	BCL-2-associated athanogene 4
ROC	Receiver operating characteristic
AUC	Area under the curve
OS	Overall survival
BCL2	B-cell lymphoma 2
TNFRSF1A	TNF receptor superfamily member 1A
HSF1	Heat shock factor 1
FADD	Fas associated via death domain
TME	Tumor microenvironment
